# Retraction Note: M2 macrophage-derived extracellular vesicles promote gastric cancer progression via a microRNA-130b-3p/MLL3/GRHL2 signaling cascade

**DOI:** 10.1186/s13046-023-02604-5

**Published:** 2023-01-24

**Authors:** Yu Zhang, Wenbo Meng, Ping Yue, Xun Li

**Affiliations:** 1 The First Clinical Medical School of Lanzhou University, Lanzhou, 730000 Gansu Province People’s Republic of China; 2 Department of Thoracic Surgery, The First Hospital of Lanzhou University, Lanzhou, 730000 People’s Republic of China; 3 Department of Special Minimally Invasive Surgery, The First Hospital of Lanzhou University, Lanzhou, 730000 People’s Republic of China; 4 Department of General Surgery, The First Hospital of Lanzhou University, No. 1, Donggang West Road, Chengguan District, Lanzhou, 730000 Gansu Province People’s Republic of China

**Retraction Note: J Exp Clin Cancer Res 39, 134 (2020)**

**https://doi.org/10.1186/s13046-020-01626-7**

The Editor-in-Chief has retracted this article. After publication it was noted that there was an overlap between the images in figures 3F, 3G and 8f. Furthermore irregularities have also been detected in figure 8C. The Editor-in-Chief has therefore lost confidence in the reliability of the results presented in this article. All authors agree to this retraction.

